# First Report in the Americas of *S. enterica* Var. Enteritidis Carrying *bla*_NDM-1_ in a Putatively New Sub-Lineage of IncC2 Plasmids

**DOI:** 10.3390/antibiotics14060620

**Published:** 2025-06-18

**Authors:** Nicolás F. Cordeiro, Romina Papa-Ezdra, Germán Traglia, Inés Bado, Virginia García-Fulgueiras, María N. Cortinas, Leticia Caiata, Mariana López-Vega, Ana Otero, Martín López, Patricia Hitateguy, Cristina Mogdasy, Rafael Vignoli

**Affiliations:** 1 Unidad Académica de Bacteriología y Virología, Instituto de Higiene, Facultad de Medicina, Av. Alfredo Navarro 3051, Montevideo 11600, Uruguay; ncordeiro@higiene.edu.uy (N.F.C.); rpapa@higiene.edu.uy (R.P.-E.); ibado@higiene.edu.uy (I.B.); virginiagarcia@higiene.edu.uy (V.G.-F.); 2 Unidad de Genómica y Bioinformática, Departamento de Ciencias Biológicas, CENUR Litoral Norte, Universidad de la República, Salto 50000, Uruguay; gtraglia@higiene.edu.uy; 3 Departamentos de Laboratorios de Salud Pública, Ministerio de Salud Pública, Montevideo 11600, Uruguay; mncortinas@msp.gub.uy (M.N.C.); lcaiata@msp.gub.uy (L.C.); mlopez@msp.gub.uy (M.L.-V.); aotero@msp.gub.uy (A.O.); cmogdasy@medicauruguaya.com.uy (C.M.); 4 Sanatorio Círculo Católico, Montevideo 11800, Uruguay; mlopez@circulocatolico.com.uy (M.L.); patricia.hitateguy@asse.com.uy (P.H.)

**Keywords:** carbapenemases, antimicrobial resistance, NDM-1, *Salmonella enterica*, transposon

## Abstract

Background: Infections caused by carbapenem-resistant Enterobacterales have steadily multiplied over time, becoming a major threat to healthcare systems due to limited therapeutic options and high case-fatality rates. Case report: We studied a patient who, after being discharged from an ICU, developed salmonellosis caused by an antibiotic-susceptible *S. enteritidis*. After undergoing treatment with ciprofloxacin, the patient presented an episode of asymptomatic bacteriuria originated by a carbapenem and ciprofloxacin-resistant *S. enteritidis*. Results: Whole genome sequencing analysis revealed that both *Salmonella* isolates belonged to the same strain, and that isolate SEn_T2 acquired a plasmid carrying both *bla*_NDM-1_ and *qnrA1* genes (pIncCSEn) which was previously present in the patient’s gut in at least one *Enterobacter cloacae* isolate. Additionally, pIncCSEN was identified as a putatively new sub-lineage of IncC2 plasmids which lacked the first copy of the methyltransferase gene *dcm* and the *rhs* gene. The resistance genes *bla*_NDM-1_ and *qnrA1* were incorporated into a Tn*21*-derived transposon that included a complex class 1 integron whose genetic arrangement was: *intI1*- *dfrA12*- *orfF*- *aadA2*- *qacEΔ1*-*sul1*-IS*CR1*- *trpF*- *ble*- *bla*_NDM-1_ (in reverse direction)- IS*Aba125*-IS*CR1*- *qnrA*- *cmlA1*- *qacEΔ1*-*sul1*. Conclusions: Antimicrobial persistence and co-selection of antibiotic resistance play an important role in the dissemination of antimicrobial resistance genes; in this regard, a joint effort involving the infection control team, effective antibiotic stewardship, and genomic surveillance could help mitigate the spread of these multidrug resistant microorganisms.

## 1. Introduction

Human salmonellosis can be divided into three main diseases, typhoid fever, paratyphoid fever and non-typhoidal *Salmonella* (NTS) infections [1]. In the United States alone, NTS is responsible for 1.35 million cases, 26,500 hospitalizations, and 420 deaths, annually (https://www.cdc.gov/salmonella/, accessed on 18 June 2024). Current guidelines indicate that only groups at increased risk for invasive NTS infections (e.g., neonates, elderly patients, persons with cardiac disease, and immunocompromised patients, etc.) should be treated [2]. Ceftriaxone, ciprofloxacin, trimethoprim-sulfamethoxazole or amoxicillin are the recommended primary treatment options for invasive salmonellosis [2]. Antimicrobial resistance in NTS is variable, and is related to factors such as serotype, source, and geographical location [3]. Accordingly, considering that the reservoir for most *Salmonella enterica* strains that cause human infections is the digestive tract of livestock, it is not surprising that the resistance profile and resistance genes present in this species resemble those detected in other enteric microorganisms present in livestock [3,4,5]. In this regard, transferable resistance to tetracycline, streptomycin, quinolones, and even oxyimino cephalosporins (mediated by ESBLs or plasmid-borne AmpC enzymes) is quite frequent [5,6].

Although carbapenems should not be administered to food-producing animals [7], in recent years the emergence of carbapemase-producing *Salmonella enterica* isolates, especially metallo-carbapenemases, is a matter of concern [8]. Among the various types of transferable metallo-β-lactamases (MBLs), NDM enzymes stand out for showing a faster and wider range of dissemination [9]; so far, the presence of this MBL has been reported in over 90 countries, including Uruguay [10,11]. Although NDM alleles have been mainly reported in Enterobacterales isolates of clinical origin, other sources include the environment [12,13], wild animals, pets, livestock [14,15] as well as food [16,17].

Several genetic platforms have been associated with *bla*_NDM_ alleles. In Enterobacterales, these genes have been identified on plasmids belonging to at least 20 different incompatibility groups, with IncX3, IncFII, and IncC being the most common [10]. Among these, IncC plasmids-broad-host-range plasmids with sizes ranging approximately 68–330 kb- have been extensively studied for their role in mobilizing extended-spectrum β-lactamase and carbapenemase genes, along with other resistance determinants [18,19]. Notably, the occurrence of *bla*_NDM_ alleles in IncC plasmids has been primarily associated with type 1a variants [20]. In our country, *bla*_NDM-1_ has been previously detected in various species within the *Enterobacteriaceae* and *Morganellaceae* families, predominantly carried by IncC plasmids [11,21].

The recent detection of a carbapenem-resistant *S. enterica* serovar Enteritidis (*S. enteritidis*) harboring *bla*_NDM-1_ on a type 2 IncC2 plasmid, in Uruguay and possibly in the Americas, represents a significant public health concern. Accordingly, our objective was to analyze said isolate, and to gain some insight into the different factors underlying the plasmid mediated dissemination of *bla*_NDM_ genes across species.

## 2. Results

### 2.1. Case Report

A 78-year-old male with a history of hypertension, type-2 diabetes mellitus, ischemic heart disease, and a stroke episode in 2021, was admitted into a moderate-care setting with radiological and antigenic diagnosis of COVID-19 pneumonitis. On day 5, he was transferred to the ICU due to worsening dyspnea which required invasive mechanical ventilation. Routine rectal swabbing in search of carbapenemase-producing pathogens yielded an NDM-producing *E. cloacae* (hereinafter Eclo_NDM). Due to prolonged stay in the ICU, the patient developed a *K. pneumoniae*-induced purulent tracheobronchitis and received ceftriaxone for 7 days. Later, the subject also developed a catheter-related bloodstream infection due to *A. baumannii*, undergoing treatment with double-dose ampicillin-sulbactam for 7 days.

On day 35, the patient was transferred from the ICU into a moderate-care room, and on day 42 developed malaise, fever, and diarrhea. Bacteriological cultures both from blood and stool samples yielded *Salmonella enterica* var. Enteritidis, isolate T1 (hereinafter SEn_T1). On day 45, antibiotic treatment with ciprofloxacin was initiated, lasting for 14 days. The patient was finally discharged on day 54.

Twenty-four days after discharge (day 82), the patient was readmitted to the Emergency Department with fever and malaise lasting 72 h. General assessment and paraclinical studies, including a bacteriological round, revealed an upper respiratory syndrome, with chest X-ray findings suggestive of acute pneumonia. Additionally, a urine culture revealed the presence of *Salmonella enterica* var. Enteritidis isolate T2 (hereafter SEn_T2). Finally, after empirical ceftriaxone treatment, the patient achieved clinical recovery.

### 2.2. Susceptibility Testing and Antibiotic Resistance Genes

SEn_T1 showed susceptibility to all the antibiotics tested. Conversely, SEn_T2 displayed resistance to trimethoprim-sulfamethoxazole, oxyimino-cephalosporins and carbapenems, while showing intermediate susceptibility to ciprofloxacin. Similarly, Eclo_NDM exhibited a similar resistance profile to SEn_T2 but was resistant to ciprofloxacin (Table 1).

Both SEn_T2 and Eclo_NDM displayed a DDST result consistent with metallo-β-lactamase production, which was confirmed as *bla*_NDM_ by PCR. The quinolone resistance gene *qnrA* was also detected in both isolates by PCR.

### 2.3. Plasmid Transfer Assays

Conjugation assays yielded positive results using either Eclo_NDM or SEn_T2 as donors. The conjugation frequency for both plasmids was ~4 × 10^−3^ (transconjugants/donors), which constitutes a high transfer efficiency for this type of plasmids (see below). Transconjugants (hereinafter designated as TcEclo_NDM and TcSEn_T2, respectively) showed resistance to carbapenems, ceftazidime and trimethoprim-sulfamethoxazole, and intermediate susceptibility to ciprofloxacin; furthermore, both transconjugants exhibited an 8-fold increase in MIC levels to ciprofloxacin, compared to the recipient strain (Table 1). DDST results were also compatible with metallo-β-lactamase production. Moreover, PCR assays confirmed the transfer of resistance genes *bla*_NDM_ and *qnrA*.

### 2.4. In Silico Analysis

After being assembled and polished, genomic DNA sequences of isolates Eclo_NDM, SEn_T1 and SEn_T2 were analyzed by online servers and standalone bioinformatic software. Carbapenem and quinolone resistance genes *bla*_NDM-1_ and *qnrA1* respectively, were detected in both Eclo_NDM and SEn_T2, confirming PCR findings. Both strains also harbored IncC and IncFIB plasmids, which will be discussed later. All other relevant data are summarized in Table 2.

### 2.5. Comparative Genomics

A genome comparison between isolates SEn_T1 and SEn_T2 revealed that both belonged to sequence-type ST11 and differed by only two mutations: one single nucleotide polymorphism (SNP) and a 777 bp insertion in the chromosome of SEn_T2. Additionally, wgMLST analysis revealed the SEn_T1 and SEn_T2 differed in 5/21,056 loci and thus belong to the same clone/strain [22]. Given that both isolates were recovered 40 days apart, these findings suggest that such minor changes between SEn_T1 and SEn_T2 likely arose within the patient’s gut.

Additionally, while both isolates carried an IncF plasmid (pIncFSEn), SEn_T2 also harbored an IncC plasmid (pIncCSEn) encoding *bla*_NDM-1_ and *qnrA1*, along with other antibiotic resistance genes. These findings suggest that SEn_T1 and SEn_T2 represent the same clone and that the occurrence of plasmid pIncCSEn in SEn_T2 could be the outcome of a horizontal genetic transfer (HGT) event between Eclo_NDM and SEn_T1.

To investigate this possibility, we conducted a comparative genomic analysis between the IncC plasmids found in Eclo_NDM and SEn_T2. The analysis revealed that both plasmids were identical, strongly supporting the hypothesis that the HGT event occurred within the patient’s gut.

### 2.6. Description of Plasmid pIncCSEn

In *S.* Enterica SEn_T2 and in *E. cloacae* Eclo_NDM, both *bla*_NDM-1_ and *qnrA1* were encoded in identical type-2 IncC plasmids, hereinafter designated pIncCSEn. Overall pIncCSEn was ∼168 kb in size, had a GC% content of 52.75 and featured 210 open reading frames. The backbone of plasmid pIncCSEn spanned 122.8 kb and harbored all the necessary genes required for plasmid replication, maintenance, partitioning and conjugal transfer.

**Figure 1:**
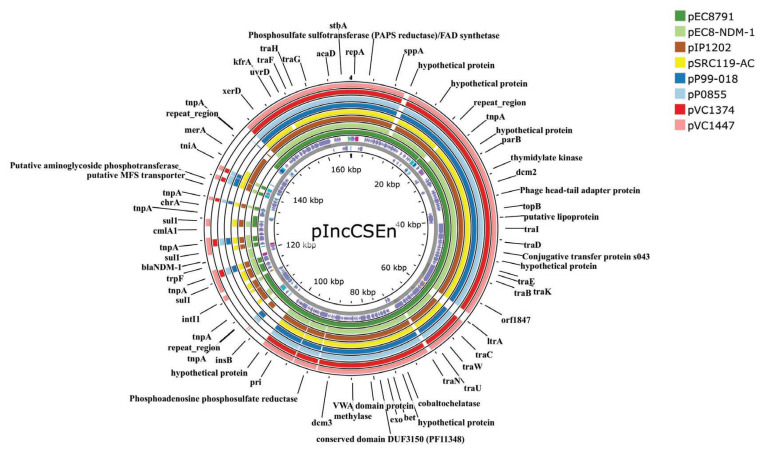
Nucleotide Identity analysis of type 2 IncC plasmids. The innermost circle corresponds to pIncCSEn. The different features are depicted in their corresponding DNA strand (violet arrows: CDS; cyan blocks: repeat regions; magenta blocks: miscellaneous features; orange blocks: miscellaneous RNA). The remaining plasmids are depicted as concentric circles. Filled blocks correspond to regions of homology. The image was generated using the Proksee webpage (https://proksee.ca/) [23].

Comparison with other type-2 IncC plasmids showed that the closest match corresponded to pEc8791 (accession *n* MZ465532), obtained from a clinical *E. coli* isolate from Argentina. In this regard, pEc8791 featured different beta-lactam and fluoroquinolone resistance genes (i.e., *bla*_PER-2_, *aac(6′)-Ib-cr*) and lacked the entire mercury resistance operon and the chromate transport-encoding gene *chrA*. The second closest match corresponded to plasmid pEC8-NDM-1 (accession *n* CP060954), obtained from a carbapenem-resistant *E. coli* clinical isolate from China. Although this plasmid also carried *bla*_NDM-1_ in a complex class 1 integron, its variable regions featured different resistance genes, suggesting a different origin for such a mobile genetic element. Furthermore, pEC8-NDM-1 lacked the mercury resistance operon and a group II intron gene (*ltrA*) downstream the plasmid transfer gene *traC*, both of which are present in pIncCSEn (Figure 1).

In contrast with typical IncC plasmids which generally feature three copies of the methyltransferase gene *dcm*, pIncCSEn is missing *dcm1.* Furthermore, the backbone section corresponding to the insertion site of ARI-A, usually located in the vicinity or within the *rhs* gene in type-2 IncC plasmids, is also absent in pIncCSEn. In this regard, close inspection of nucleotide sequences of other type-2 IncC plasmids, showed that plasmids pEc8791, and pEc61B (accession *n* CP053105) also lacked *rhs*. Interestingly, both plasmids were described in our neighboring countries Argentina and Brazil, respectively, albeit harboring completely different resistance islands.

**Figure 2:**
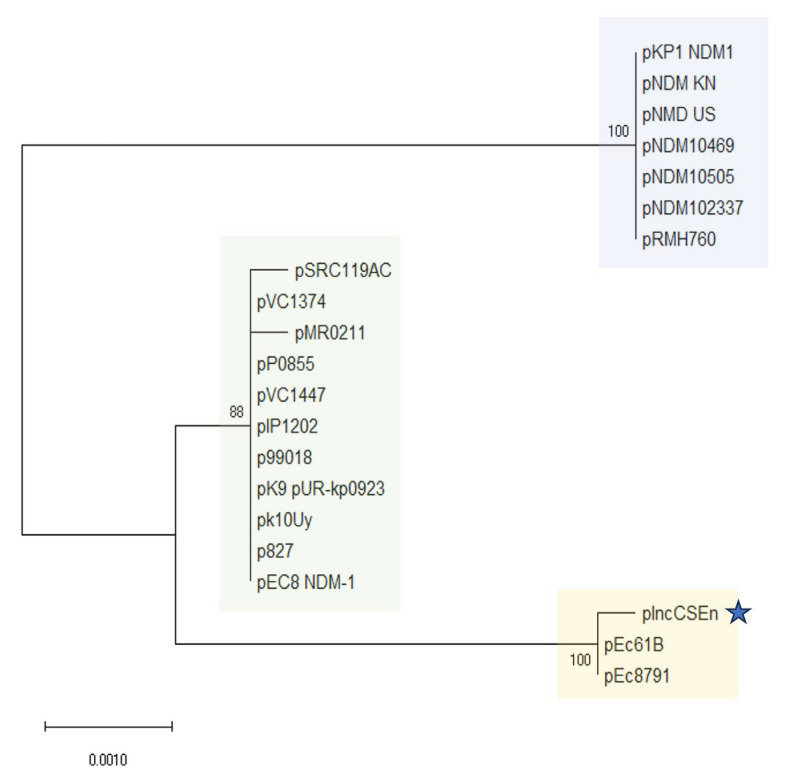
Evolutionary relationships of IncC plasmids. The percentage of replicate trees in which the associated taxa clustered together in the bootstrap test (500 replicates) are shown next to the branches. The tree is drawn to scale, with branch lengths in the same units as those of the evolutionary distances used to infer the phylogenetic tree. The blue-shaded box corresponds to type-1 IncC plasmids, the green-shaded box corresponds to type-2 IncC plasmids, and the yellow-shaded box corresponds to a putative sub-lineage of IncC plasmids (the star indicates the plasmid described in this work).

Furthermore, we conducted a phylogenetic analysis of the nucleotide sequences of the alleles employed in the IncC pMLST scheme (i.e., *repA*, *parA*, *parB*, A053). In this sense, the resulting dendrogram showed that plasmid pIncCSEn, clustered with both pEc8791 and pEc61B, forming an offshoot of the type-2 IncC clade (Figure 2).

### 2.7. Resistance Region

Like other type-2 IncC plasmids, pIncCSEn featured two resistance islands (ARI-A and ARI-B). The latter was inserted upstream of plasmid stability genes *parAB* and consisted of sulfonamide resistance gene *sul2* preceded by a phosphoglucosamine mutase gene and IS*CR2*. Conversely, ARI-A spanned ~42.3 kb and encoded multiple ARGs. It was inserted between the DNA primase gene (*pri*) and an integrase/recombinase gene (*xerD*) and was constituted by a new Tn*21*-derived transposon designated Tn*8710* (see below), whose IRL and IRR were interrupted by two copies of IS*4321*-like. Accordingly, Tn*7810* was 39269 bp and was bracketed by DRL and DRR direct repeats (5’-TAATA-3’). Additionally, it was flanked by two copies of IS*1*-family insertion sequences, namely IS*1R* and IS*1X4*-like, at the left and right flank respectively. Additionally, adjacent to IS*1R* there was the chloramphenicol resistance gene *catA1*.

**Figure 3:**
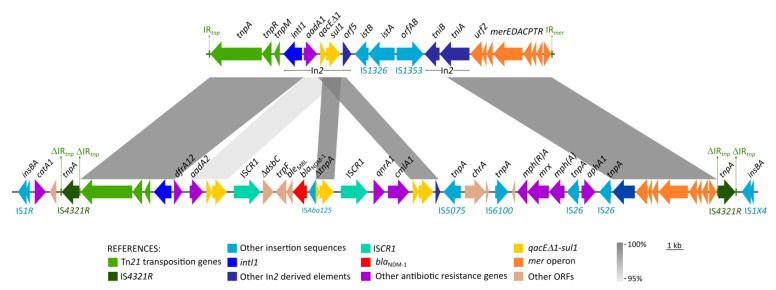
Linear map of ARI-A in pIncCSEn, compared with the original structure of Tn*21*. Homologous segments are shown as grey blocks as indicated in the reference.

The remnants of Tn*21* that are part of Tn*8710* in plasmid pIncSEn are divided into two fragments and will be described in accordance with the sequence and structure reported by Liebert et al. (accession number AF071413) [24]. The left segment consists of the transposition elements of Tn*21*, comprising *tnpA*, *tnpR*, *res* sites I, II and III, and *tnpM*, followed by class 1 integron integrase gene *intI1*. Unlike In*2*, which carries *aadA1* as gene cassette, the class 1 integron associated with Tn*8710* carries *dfrA12* and *aadA2* in its variable region. On the other hand, the right segment of Tn*2*, conserved in Tn*8710*, includes a fragment of the Tn*21*-*tniA* gene, followed by the *mer* operon (*merEDACPTR*). Moreover, the central region of Tn*21*, comprising the class 1 integron accessory genes (*orf5*, *tniB* and part of *tniA*) and IS*1326*, IS*1353*, is missing in Tn*8710*, likely as the result of several insertion/deletion events probably occurred instead (Figure 3).

The class 1 integron featured by Tn*8710* was associated with a tandem of two copies of IS*CR1* and their respective variable regions, thus constituting a complex class 1 integron. Accordingly, the first variable region consisted of *dfrA12-orfF-aadA2*, and was followed by the *qacEΔ1* and *sul1* tandem; the latter were continued by the first copy of IS*CR1* interrupting the gene *dsbC*, along with *trpF*, *ble*, *bla*_NDM-1_ (in reverse direction) and *Δ*IS*Aba125*. This IS was truncated by the insertion of a second IS*CR1* followed by the genes *qnrA1*-*cmlA1* and another copy of *qacEΔ1-sul1.* BLASTn analysis of this structure yielded only partial matches, suggesting that this constitutes a novel genetic environment for *bla*_NDM-1_.

Downstream this complex class 1 integron, Tn*8710* carried other mobile elements, including IS*5075*-like in reverse orientation associated with the *chrA* gene, and IS*6100* followed by the macrolide resistance gene *mph(A)* and its regulators *mphR(A)* and *mrx*. Further downstream we detected Tn*4352*, formed by two copies of IS*26* flanking the kanamycin and neomycin resistance gene *aphA1*.

Overall, the whole resistance island encompassed multiple genes conferring resistance to several antibiotics, including β-lactams (*bla*_NDM-1_), quinolones (*qnrA1*), aminoglycosides (*aadA2*, *aphA1*), trimethoprim- sulfamethoxazole (*dfrA1* and *sul1*), chloramphenicol (*catA1*, *cmlA1*) and macrolides [*mph(A)-mphR(A)*-*mrx*]; moreover, this island also harbored resistance determinants to other antimicrobial compounds such as mercury (*mer* operon), quaternary ammonium (*qacEΔ1*) and bleomycin (*ble*).

## 3. Discussion

The success of NDM enzymes (in terms of global widespread) seems to be associated with the intracellular localization of these MBLs. In this regard, contrary to other soluble periplasmic metallo-β-lactamases, NDM is a lipoprotein that remains anchored to the inner leaflet of the outer membrane in Gram negative bacteria [25]. This peculiar localization serves two distinct functions; on the one hand, it prevents apo-enzymes (i.e., Zn^2+^-devoid NDM) from being degraded since soluble variants of NDM are less stable. On the other hand, another benefit of being anchored to the outer membrane is the secretion of NDM in outer membrane vesicles (OMV), which in turn, relieves the stress in the periplasmic space [26], and protects these enzymes against extracellular proteases and chelating agents [27]. Furthermore, these NDM-containing OMV can also alter the antimicrobial resistance profile of other bacterial populations [28].

Previous studies have suggested that the production of class B β-lactamases entailed a fitness reduction in *S. enterica*, which in turn could alter the ability of this microorganism to colonize the human gut; such biological cost could also explain the low frequency of MBL-producing *Salmonella* isolates [29]. Nevertheless, Lopez et al. argued that NDM enzymes appear to be molded by evolution to avoid imposing a biological cost on their microbial hosts, thus resulting in a rapid and widespread dissemination of these MBLs [26]. Furthermore, several authors have pointed out that *bla*_NDM_-harboring plasmids are stably maintained even in absence of selective pressure (i.e., antibiotics) [30,31].

Although NDM-producing *Salmonella enterica* isolates have already been reported [32,33,34], the presence of *bla*_NDM-1_ in *S. enteritidis* is exceptional. Interestingly, Beukers et al. described the dissemination of a *bla*_NDM-1_-carrying plasmid among different isolates of different *Enterobacteriaceae* species (including an isolate of *S. enteritidis*); nevertheless, the authors were unable to obtain the susceptible receptor isolate (prior to the acquisition of the resistance plasmid) or to disclose the prescribed antibiotics that resulted in the selection or co-selection of such resistant microorganisms [34]. One of the strengths of our work is that it documents, step-by-step, the different factors, including the various selection and co-selection processes that may have ultimately led to the emergence of the carbapenem resistant SEn_T2 strain. Furthermore, to the best of our knowledge, this is also the first report of an NDM-1-producing *S.* Enteritidis isolate in the Americas.

As previously stated, numerous plasmid incompatibility groups have been associated with *bla*_NDM_ genes in Enterobacterales, including type 1 and 2 IncC plasmids [35]; besides having a broad host range, these mobile genetic elements have been frequently associated with the dissemination of resistance genes within Enterobacterales [36]. In this context, the 122.8 kb backbone of plasmid pIncCSEn was slightly shorter than similar plasmids previously reported by our group [37], however it harbored the necessary genes for plasmid replication, conjugal transfer and partitioning. Among the typical IncC features, pIncCSEn lacked *dcm1*, probably on account of an ARI-B-related deletion, an event already described with no deleterious consequences for the plasmid [35]. Another absent element was the *rhs* gene, a common ARI-A insertion site. However, its absence does not appear to be detrimental to the plasmid, as it constitutes a hotspot for the insertion of resistance islands. Additionally, partial *rhs* deletions have been previously reported in type-1 IncC plasmids without adverse effects [38]. In the case of pIncCSEn, the resistance island was inserted upstream of the integrase/recombinase gene *xerD*, also defined as a hotspot for antibiotic resistance island site in type 2 IncC plasmids [35].

Almost every *bla*_NDM_-carrying IncC plasmid described so far belongs either to ST1 or ST3 in the pMLST scheme [10], however, pIncCSEn could not be assigned to a specific sequence type on account of variations in genes *parB2* and *repA4.* This, in conjunction with the absence of *rhs2* suggests that pIncCSEn (along with pEC8791 and pEc61B) could belong to a new sub-lineage of IncC plasmids. However, more studies are required to corroborate such a statement.

The *bla*_NDM-1_ gene present in pIncCSEn, was embedded in a multidrug resistance island, consisting of a novel Tn*21*-derived transposon designated Tn*7810*. The Tn*21*-family has classically been involved in the accumulation and dissemination of antibiotic resistance genes [24]. On the other hand, *bla*_NDM_ has been found associated with a variety of genetic contexts, which usually involve diverse genetic elements such as insertion sequences and composite transposons. This gene has been found associated with the IS*CR1* element, mainly as part of complex class 1 integrons, along with other antibiotic resistance genes, as those described in this work [39]. In this regard, the complex class 1 integron harboring *bla*_NDM-1_ described in the present study, constitutes a novel genetic arrangement which includes *dfrA12-aadA2*, *qnrA1*, *cmlA1* and *sul1* in a single platform. Noticeably, this resistance island contains resistance genes to all the usually prescribed antibiotics for invasive non-typhoidal salmonellosis, i.e., ampicillin, third-generation cephalosporins (*bla*_NDM-1_), fluoroquinolones (*qnrA1*), trimethoprim-sulfamethoxazole (*dfrA12*, *sul1*), chloramphenicol (*catA1*, *cmlA1*), and azithromycin [*mph(A)*] [40].

The Tn*21*-terminal inverted repeats in Tn*7810* were interrupted by IS*4321R*. This insertion sequence, along with IS*4321L* and IS*5075*, is usually found inserted in the same position within Tn*21*-family transposons, impairing their capability for mobilization [41]. However, the excision of IS*4321* may restore the inverted repeats, allowing the Tn*21* transposition [42]. On the other hand, besides this Tn*21*-derived structure, other putative composite transposons were detected, flanked by homologous or identical insertion sequences such as IS*4321R* and IS*5075*, or IS*26* and IS*6100*. These elements may be able to mobilize *bla*_NDM-1_ and other antibiotic resistance genes by forming cointegrates, promoting multiple gene transfer and co-selection [39]. The complex structure displayed by Tn*7810* makes it difficult to trace the probable genetic events that originated such genetic element; in this regard, *bla*_NDM-1_ was probably mobilized by a transposon along with IS*Aba125* and then “captured” by the complex class 1 integron, in association with IS*CR1*. Other events probably involved the insertion of IS*4321R* in the Tn*21* terminal repeats, and the acquisition of other insertion sequences or composite transposons.

Finally, the detection of an NDM-1-producing *S.* Enteritidis in the Americas highlights the ongoing evolution of carbapenem resistance in Enterobacterales. Our findings reveal a probably new IncC sub-lineage carrying *bla*_NDM-1_ within a novel Tn*21*-derived transposon, emphasizing the role of mobile genetic elements in resistance dissemination.

The detailed reconstruction of selection and co-selection events, fueled by usage of critically important antibiotics, provides key insights into the stability and spread of *bla*_NDM-1_, thus reinforcing the need for improved antimicrobial stewardship, and continuous genomic surveillance to track and mitigate the expansion of carbapenem resistance, and antimicrobial resistance in general. Additionally, this case took place during the COVID-19 pandemic, a period marked by significant strain on healthcare systems. Among several contributing factors, the increased workload of infection control teams (focused primarily on limiting the spread of COVID-19 within medical facilities) led to a reduced attention to the prevention and surveillance of other infectious diseases [43]. For instance, although the patient received controlled feeding during an extended hospital stay, and bearing in mind that no other cases were detected during the same period, it is plausible that the source of his hospital-acquired salmonellosis was the introduction of contaminated food or snacks, potentially brought in by relatives or visitors following the patient’s transfer to a moderate care unit.

## 4. Materials and Methods

### 4.1. Patient

The patient was a 78-year-old male admitted to a moderate-care setting with diagnosis of COVID-19. Clinical data were collected retrospectively by reviewing the medical records. The patient signed an informed consent allowing access to such data.

### 4.2. Strains, Identification, and Antibiotic Susceptibility

Bacterial identification was performed by matrix-assisted laser desorption ionization-time-of-flight (MALDI-TOF) mass spectrometry (Bruker, Billerica, MA, USA). Antibiotic susceptibility was determined using the Vitek 2 system (bioMérieux, Marcy l’Étoile, France).

Phenotypic detection of metallo-β-lactamases, class A carbapenemases and extended-spectrum β-lactamases were performed by double-disk synergy tests (DDST) with the corresponding combination of antimicrobial agents and specific inhibitor disks [44]. Minimum inhibitory concentration assays were carried out and interpreted according to CLSI guidelines [45]. Carbapenemase genes and plasmid mediated quinolone resistance genes were sought by PCR as previously described [11].

### 4.3. Plasmid Transfer

Plasmid transfer was assessed by conjugation assays, using *E. coli* J53-2 (*pro met* Rif^r^ Nal^r^) as recipient. Briefly, log-phase LB broth cultures of recipient and donor strains were mixed in a 10:1 ratio and then incubated statically overnight at 37 °C [46]. Transconjugants were selected on Luria-Bertani agar plates supplemented with ceftazidime (2 µg/mL) and rifampicin (150 µg/mL), and transfer of *bla*_NDM_ and *qnrA* was verified by PCR [11] Conjugation frequencies were determined according to Rozwandowicz M et al. [47].

### 4.4. Whole Genome Sequencing

Genomic DNA was extracted with the NZY microbial gDNA Isolation kit, following the manufacturer’s instructions (NZYTech Genes & Enzymes, Lisbon, Portugal). DNA quality was assessed using a NanoDrop 1000 spectrophotometer (Thermo Fisher, Wilmington, DE, USA), and later quantified with Qubit^®^ 3.0 fluorometer and the dsDNA HS Assay Kit (Thermo Fisher Scientific, Waltham, MA, USA).

Illumina libraries were prepared with the Nextera XT kit (Illumina Inc., San Diego, CA, USA), and later sequenced using an Illumina MiniSeq device with a MiniSeq High-output reagent kit (Illumina Inc., San Diego, CA, USA) and a 2 × 151 bp paired-end strategy. Conversely, Nanopore libraries were prepared using the Rapid Sequencing Kit-SQK-RAD004, following the manufacturer’s instructions; libraries were later loaded onto R.9.4.1 flow cells (FLOMIN106) and sequenced (singleplex) for 10 h on a MinION Mk1B device (Oxford Nanopore Technologies, Oxford, UK). Basecalling was performed with the standalone Guppy version 6.5.7 (https://community.nanoporetech.com) using the high-accuracy model.

### 4.5. Genome Assembly

We obtained 1,998,218 Illumina reads for strain Sen_T1 (296 Mb, Cov 62,8), and 1,265,878 reads for strain Sen_T2 (183 Mb, Cov 37,5), with a Q30 = 92.2. Illumina reads were then filtered with the Fastp software v0.23.2 [48]. On the other hand, we obtained Nanopore reads equivalent to 3,2 Gb bases, with a depth ≈ 300X, for both strains, (mean read quality 7,92 (SEn_T1), and 13.2 (SEn_T2)). Accordingly, Nanopore reads were filtered using Filtlong v0.2.1 (https://github.com/rrwick/Filtlong, accessed on 1 November 2023), to remove reads <1000 bp and reads with a mean quality score <95. Genome hybrid assembly (using short and long reads) was carried out using the Trycycler software v0.5.3 along with Flye v2.9-b1768, Canu v2.2, Raven v1.8.1 and Unicycler v0.5.0 for subsample assembly [49,50,51,52]. The assembled consensus genomes were first polished with Medaka v1.6.1 (https://github.com/nanoporetech/medaka, accessed on 16 June 2024) and later with Polypolish v0.5.0 [53].

### 4.6. In Silico Analysis

Serotype prediction of *Salmonella enterica* isolates was done in silico with SeqSero2 v1.3.1 pipeline using the corresponding genome assemblies [54]; furthermore, the results were double checked by tetra correlation search using the JSpeciesWS web service (https://jspecies.ribohost.com/jspeciesws/#analyse, accessed on 16 June 2024). On the other hand, prediction of antimicrobial resistance genes was carried out using the AMRFinderPlus software v3.11.18, with default parameters [55], whereas mobile genetic elements (i.e., plasmids) were detected with ABRicate v1.01 using the PlasmidFinder database (selection criteria-minimum coverage: 70%, minimum identity: 70%) (https://github.com/tseemann/abricate, accessed on 1 November 2023). Multilocus sequence-typing for both *S. enterica* isolates and the *E. cloacae* strain, and pMLST were predicted with the MLST 2.0 (https://cge.food.dtu.dk/services/MLST/, accessed on 16 June 2024) and the pMLST 2.0 suites, respectively (https://cge.food.dtu.dk/services/pMLST/, accessed on 16 June 2024). Furthermore, wgMLST profiles for both *Salmonella enterica* isolates were obtained using the Enterobase v1.2.0 suite (https://enterobase.warwick.ac.uk/species/index/senterica, accessed on 11 June 2025); in this sense, long and short fastq reads of both isolates were uploaded onto and assembled using the Enterobase webpage, and profiled using a 21,056-loci scheme. Conversely, genome annotation and plasmid annotation using the RAST 2.0 webpage [56] and manually curated with the Artemis software [57]. Finally, identification of putative orthologs and pangenome analysis was performed with the Roary v.3.11.2 package [58], whereas mutation assessment (i.e., detection of SNPs and/or insertions/deletions) was carried out with the breseq/gdtools v0.38.1 pipeline, using the default parameters [59].

Comparisons with nucleotide sequences available in public databases were performed with BLASTn v2.16.0 (https://blast.ncbi.nlm.nih.gov/Blast.cgi, accessed on 15 July 2024), and physical maps were generated using EasyFig 2.1 and the Proksee-Genome Analysis web tool (https://proksee.ca/).

Raw Fastq sequences were deposited in Genbank under Bioproject accession number PRJNA950342.

### 4.7. Phylogenetic Analysis

Nucleotide sequences of genes *repA*, *parA*, *parB* and locus A053 were extracted, and conjoined, from plasmids publicly available online. Sequence alignment, and dendrogram were performed with MEGA X [60]. In this regard, the evolutionary history was inferred using the Neighbor-Joining method, with a 500-replicate bootstrap test [61,62]; the evolutionary distances were computed using the Maximum Composite Likelihood method considering the number of base substitutions per site [63].

## Figures and Tables

**Table 1:** Minimum inhibitory concentrations (in mg/L) and its interpretation according to CLSI 2023 (33rd Edition) breakpoints, for clinical strains, transconjugants and receptor strain.

	*E. cloacae* Eclo_NDM	*S. enteritidis* SEn_1	*S. enteritidis* SEn_2	*E. coli* TcEclo_NDM	*E. coli* TcSEn_2	*E. coli* J53-2
Ampicillin-Sulbactam	-	≤2 (S)	≥32 (R)	≥32 (R)	≥32 (R)	≤2 (S)
Piperacillin-Tazobactam	≥128 (R)	≤4 (S)	64 (R)	≥128 (R)	≥128 (R)	≤4 (S)
Ceftazidime	≥64 (R)	0.25 (S)	≥64 (R)	≥64 (R)	≥64 (R)	0.5 (S)
Ceftazidime-Tazobactam	≥16 (R)	≤0.12 (S)	≥16 (R)	≥16 (R)	≥16 (R)	≤0.12 (S)
Ceftolozane-Tazobactam	≥32 (R)	≤0.25 (S)	16 (R)	≥32 (R)	≥32 (R)	≤0.25 (S)
Cefepime	≥32 (R)	≤0.12 (S)	16 (R)	0.5 (S)	0.5 (S)	≤0.12 (S)
Aztreonam	≥64 (R)	≤1 (S)	≤1 (S)	≤1 (S)	≤1 (S)	≤1 (S)
Ertapenem	≥8 (R)	≤0.12 (S)	≥8 (R)	≥8 (R)	≥8 (R)	≤0.12 (S)
Imipenem	8 (R)	≤0.25 (S)	8 (R)	8 (R)	4 (R)	≤0.25 (S)
Meropenem	≥16 (R)	≤0.25 (S)	≥16 (R)	8 (R)	8 (R)	≤0.25 (S)
Amikacin	≤1 (S)	≤1 (S)	≤1 (S)	≤1 (S)	≤1 (S)	≤1 (S)
Ciprofloxacin	≥4 (R)	≤0.06 (S)	0.25 (I)	0.5 (I)	0.5 (I)	≤0.06 (S)

R, resistant; S, susceptible; I, intermediate.

**Table 2:** Data of the microorganisms discussed in this work.

	Eclo_NDM	SEn_T1	SEn_T2
AMR genes	*aadA2*, *aph(3*’*)-Ia*, *sul1*, *sul2*, *dfrA12*, *qnrA1*, *qacE∆1*, *catA1*, *cmlA1*, *bla*_NDM-1_*fosA*, *bla*_ACT-7_,	*aac(6*’*)-Iaa*	*aadA2*, *aph(3*’*)-Ia*, *sul1*, *sul2*, *dfrA12*, *qnrA1*, *qacE∆1*, *catA1*, *cmlA1*, *bla*_NDM-1_*aac(6*’*)-Iaa*,
Plasmid incompatibility groups	IncC, IncFIB, IncR	IncFIB	IncC, IncFIB
MLST	ST146	ST11	ST11
wgMLST	NA	ST733989	ST733988
pMLST	IncFIB:ST NT IncC: ST3,9 *	IncFIB: ST_22	IncFIB: ST_22 IncC: ST3,9 *
Serovar	NA	Enteritidis	Enteritidis

* Both IncC plasmids show identical point mutations in genes *parB2* and *repA4*; numbers indicate the nearest ST matches. NT: Not typeable. NA: Not applicable.

## Data Availability

Raw Fastq sequence are openly available in Genbank under Bioproject accession number PRJNA950342 (https://www.ncbi.nlm.nih.gov/bioproject/PRJNA950342/ (accessed on 16 June 2024)), and in Enterobase with the same accession number (https://enterobase.warwick.ac.uk/species/index/senterica (accessed on 16 June 2024)).
